# Assembly of Linear Nano-Chains from Iron Oxide Nanospheres with Asymmetric Surface Chemistry

**DOI:** 10.1371/journal.pone.0015927

**Published:** 2011-01-06

**Authors:** Pubudu M. Peiris, Erik Schmidt, Michael Calabrese, Efstathios Karathanasis

**Affiliations:** Department of Biomedical Engineering and Department of Radiology, Case Center for Imaging Research, Case Western Reserve University, Cleveland, Ohio, United States of America; University of Pennsylvania, United States of America

## Abstract

Besides the multifunctionality, another equally important aspect of nanoparticles is their engineerability to control the geometrical and chemical properties during fabrication. In this work, we exploited this aspect to define asymmetric surface chemistry of an iron oxide nanosphere by controlling the topology of ligand expression on its surface resulting in a particle with two faces, one displaying only amines and the other only thiols. Specifically, amine-functionalized iron oxide nanospheres were attached on a solid support via a crosslinker containing a disulfide bridge. Liberation of the nanosphere using thiolytic cleavage created thiols on the portion of the particle's surface that interacted with the solid support. Employing a solid-phase strategy and a step-by-step addition of particles, the two unique faces on the same nanosphere served as fittings to assemble them into linear nano-chains. Assembly of chains with various lengths and aspect ratios was controlled by the size and number of the added nanospheres. The characteristics of those chains showed a high degree of uniformity indicating the exceptional control of the synthetic process. Notably, one of the unique properties of the iron oxide nano-chains was an increased magnetic relaxivity, indicating their potential use as contrast agents for magnetic resonance imaging.

## Introduction

Nanoparticles [1], [2] are excellent delivery vehicles for therapeutic and imaging agents with improved biodistribution and increased delivery efficiency to solid tumors [3], [4]. In particular, nanomedicine's greatest advantage over conventional therapies is its ability to combine more than one function by enabling the design of multifunctional nanoparticles that target, image, and destroy tumors [5]. This has led to the development of various nanoparticle delivery systems such as liposomes, dendrimers, other lipidic and polymeric nanoparticles, and metal nanoparticles (e.g. iron oxide and gold) [6]. While the shape of the majority of these particles is spherical due to the methods of preparation, recent advances have fabricated oblate- and rod-shaped nanostructures suitable for biomedical applications such as gold nanorods [7], gold nanochains [8], nanoworms [9], [10], and nanonecklaces [11]. For example, the so-called nanoworms consist of iron oxide cores aligned along strands of high-molecular weight dextran [10]. A nanonecklace was formed by attaching monofunctionalized gold nanoparticles onto polylysine [11].

Interestingly, the shape at the nanoscale has been shown to influence the *in vivo* performance of nanomaterials. In recent computational and experimental studies, it has been shown that the particle size and shape play a central role in the transport of nanoparticles in the abnormal tumor microcirculation [12], [13], [14], [15]. Contrary to spheres that move along the center of a vessel in microcirculation, oblate-shaped particles can drift laterally moving in close proximity to the endothelium [16]. This allows the particle to interact with the vessel walls to either target vascular specific biomarkers or extravasate through the tumor leaky endothelium into the tumor interstitium. Furthermore, oblate-shaped particles decorated with ligands targeting overexpressed cellular biomarkers (e.g. receptors) can display stronger binding due to the large portion of the particle's surface available for interaction with the cell surface [17], [18].

**Figure 1:**
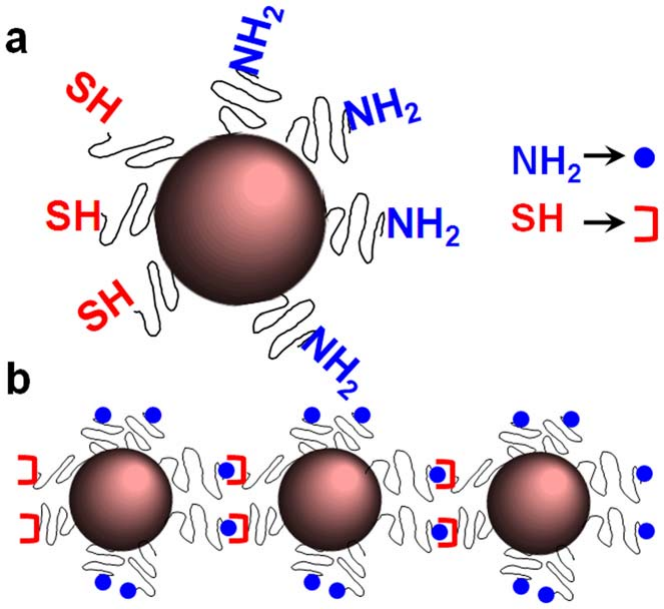
Illustration of nanospheres with two distinctive faces in terms of chemical functionality and their use as components of nano-chains. (a) Nanospheres with asymmetric surface chemistry (ASC) and (b) linear nano-chains assembled from spheres with ASC.

In this work, we exploited the engineerability of nanoparticles to shape them with defined geometrical and chemical properties. Firstly, the chemical properties of a nanosphere were defined by controlling the topology of functional groups on its surface. Assuming attachment of a nanosphere decorated with one type of functional group on a solid surface via a cleavable crosslinker, liberation via cleavage can result in a new functional group located at the portion of the nanosphere's surface that interacted with the solid surface. For example, thiolytic cleavage of a crosslinker containing a disulfide bridge will create a thiol group. More specifically, solid-phase chemistry was used to partially convert amine groups on the surface of iron oxide nanospheres into thiols (Figure 1a) resulting in a particle with asymmetric surface chemistry (ASC). Other groups have reported nanoparticles with bifunctional surfaces. Gold nanospheres with a single functional group on their surface have been synthesized using a solid-phase place exchange reaction [19], [20], [21]. Sardar et al. reported asymmetric functionalization of gold nanoparticle via solid phase synthesis using a silane functionalized glass surface based on the inaccessibility of the “replacing” group to reach the region of the nanoparticles adsorbed on the substrate [22]. Furthermore, nanospheres with two distinct phases, termed Janus nanoparticles, have been reported [23], [24], which require the fusion of different polymer matrixes. It should be noted that the ASC strategy reported here provides exceptional flexibility in controlling the surface functionalization that can be employed to various types of nanoparticles (e.g. liposome, dendrimer, metal particle), since the synthetic method is carried out in aqueous environment and physiological pH.

Using a step-by-step addition of particles and solid-phase chemistry, the two unique faces on the nanoparticle served as fittings to assemble the particles with ASC into nano-chains in a controlled manner (Figure 1b). Different nano-chains were synthesized and characterized consisting of nanospheres with different sizes. The nano-chain exhibited a significant increase in T2 relaxivity compared to its constituting iron oxide nanospheres, which implies that they can be potent imaging agents for magnetic resonance imaging (MRI).

## Results

### Synthesis of nanoparticles with asymmetric surface chemistry

**Figure 2:**
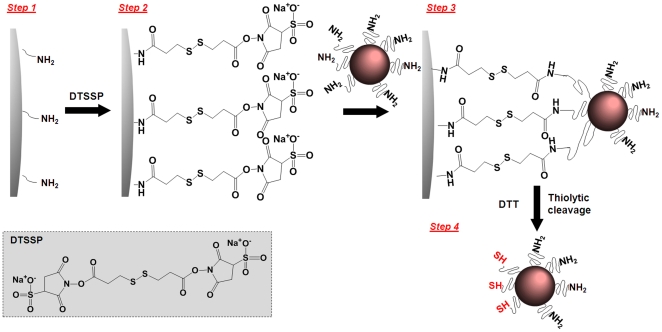
Reaction scheme of the fabrication of nanospheres with asymmetric surface chemistry (ASC) showing the partial modification of the functional groups on a nanoparticle's surface using solid-phase chemistry.

Solid phase synthesis was used to produce iron oxide nanospheres with ASC consisting of two areas with distinct functional group distribution (i.e. thiols and amine) as shown in Figure 2. We used the CLEAR resin which is an amine-functionalized resin particle of 100 µm with a swelling in water of ∼5.5 ml/g, and a substitution level of 0.72 mmol/g (manufacturer's product specifications sheet). Using a previously published method [19], [20], we calculated 1.172×10^9^ amines per bead of resin. As illustrated in Figure 2, we used large excess of homobifunctional cleavable cross-linkers such as DTSSP to react all the amines on the resin. As a result, the solid-phase surface displayed multiple active sites available for binding to the upcoming nanospheres. Once the amine-functionalized iron oxide nanospheres were attached to the solid support via DTSSP, the disulfide bond in DTSSP could be easily cleaved with the addition of a mild reducing agent such as TCEP (or DTT). As a result of this cleavage, the portion of the nanosphere's surface that interacted with the solid support was decorated with thiol groups whereas the rest of the surface maintained its initial decoration of amine groups. We modified the surface of three iron oxide nanospheres with sizes of 10, 20, and 30 nm.

**Figure 3:**
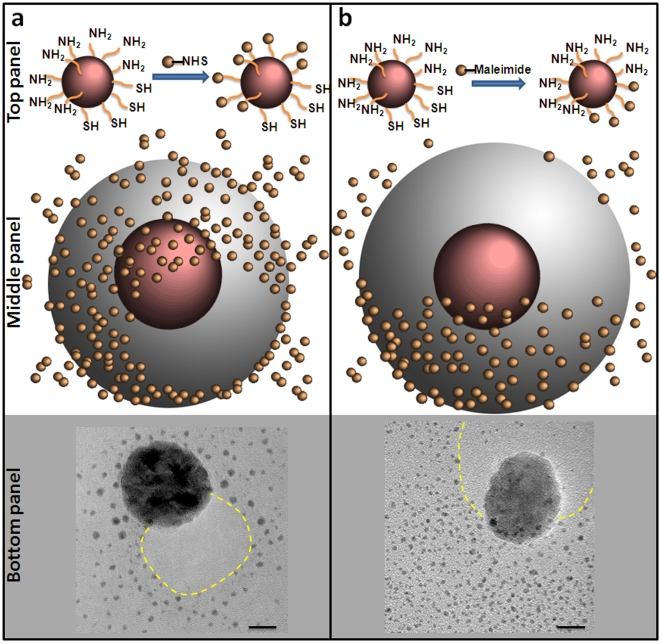
TEM images reveal the asymmetric surface chemistry of iron oxide nanospheres. (a) TEM image (bottom panel) of an iron oxide nanosphere with an asymmetric surface chemistry displaying a controlled expression of amines and thiols on its surface. Top panel shows an illustration of a 1.4 nm gold probe (AuNP) that was used to tag the amines on the surface of the iron oxide nanosphere. using NHS-AuNP. The middle panel shows a cartoon of the iron oxide particle decorated with the AuNP tags. (b) Similarly, maleimide-AuNP was used to decorate the thiols on the surface of the iron oxide particle. Dotted line in yellow indicates the approximate location of the polymer surface with the modified functional group. Scale bar is 10 nm.

The distribution and number of thiols and amines on the nanosphere's surface were evaluated qualitatively using transmission electron microscopy (TEM), theoretically, and quantitatively using fluorescence-based assay (*vide infra*). TEM evaluation of the topology of each functional group was achieved by tagging the amines or the thiols on the nanoparticle surface with an excess of the appropriate gold probe (diameter of 1.4 nm) as shown in Figure 3. More specifically, we incubated the ASC nanospheres (30 nm in diameter) with (a) NHS-functionalized gold probes reactive towards amine groups (Figure 3a; top panel), (b) maleimide-functionalized gold probes reactive towards thiol groups (Figure 3b; top panel), (c) non-functionalized gold probes as a negative control (image not shown), and (d) a mixture of NHS- and maleimide- functionalized gold probes as a positive control (image not shown). In the two latter cases, the nanosphere had either no gold probes in its proximity (condition c) or gold probes distributed homogeneously everywhere (condition d). It should be noted that each TEM image is a 2D summation of an iron oxide nanosphere with the iron core being about 30 nm and the polymer coating being about 10 nm as the sketches illustrate (Figure 3a and b; middle panel). Figure 3a (bottom panel) shows that a portion of the nanosphere's surface could not be tagged by the NHS-functionalized gold probe, indicating the absence of amines. In contrast, a smaller portion of the nanosphere's surface could be tagged by the maleimide- functionalized gold probe (Figure 3b; bottom panel). A visual inspection of the two TEM images implies that about 70% and 30% of the sphere's surface contains amines and thiols, respectively. TEM images of multiple ASC particles have been obtained at different magnifications. We observed that the vast majority of the particles were modified displaying a similar surface asymmetry. However, we elected to display a TEM imaging with one particle at a very high magnification to clearly show the ASC modification.

**Figure 4:**
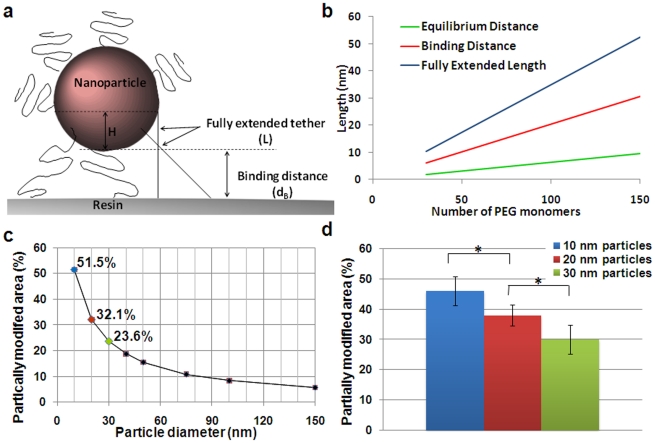
Theoretical and experimental analysis of the asymmetry on the nanosphere's surface chemistry. (a) Schematic illustration of nanoparticle-resin surface binding. (b) Length of PEG polymers with respect to tether interactions as a function of repeating monomers per PEG molecule. The equilibrium distance is defined as the distance corresponding to the Flory radius of a polymer. The binding distance is defined as the length of polymer at which maximum resin-ligand complexes are formed. The fully extended length corresponds to the maximum length of a polymer tether. (c) Theoretical estimation of the partially modified area (PMA) as a function of nanoparticle size. (d) Experimental measurement (n = 3) of the portion of amines modified to thiols using NHS-functionalizedAlexa-488 to fluorescently tag the amines (* indicates p<0.05; data presented as mean ± standard deviation).

To study the effect of the nanosphere's diameter and tether length on the surface modification (as shown in Figure 4a), the partially modified area (PMA) was calculated [25], [26]. The PMA of the nanosphere is defined as the fraction of the surface that is capable to bind to the solid support. Using a combination of Monte Carlo simulations and diffusion reaction theory, Jeppesen et al [27] have demonstrated that the binding distance can be approximated as a function of the tether size. For a polymer like polyethylene glycol (PEG), used as a coating of the iron oxide nanosphere, Figure 4b shows a plot of binding distance as a function of PEG monomers as extrapolated from Jeppesen et al [27]. The binding distance is higher than the equilibrium length of PEG, which is proportional to the Flory radius, and smaller than the maximum extended length of the tether. As shown in Figure 3c, the PMA is 51.5, 32.1 and 23.6% for a 10, 20 and 30 nm sphere, respectively.

The theoretical design of the ASC nanospheres was validated experimentally using a modification of a previously published fluorescence-based assay [28]. Using Alexa-488-NHS ester to tag the amines on the nanosphere's surface, the fluorescence intensity of the ASC nanospheres was compared to the parent, non-modified nanospheres (only surface amines). The difference in the fluorescence measurements between the ASC and parent spheres is indicated as “modified surface amines” (i.e. amines converted to thiols) in Figure 4d. These data clearly show the relationship between the size and the surface modification. Since this method takes under account the total number of amine groups on the surface of the nanospheres in the entire suspension, the estimated “modified surface amines” might not be an accurate measure of the modification of each individual particle. However, the fluorescence-based measurement is in fairly good agreement with the theoretical estimation.

### Preparation of linear nano-chains

**Figure 5:**
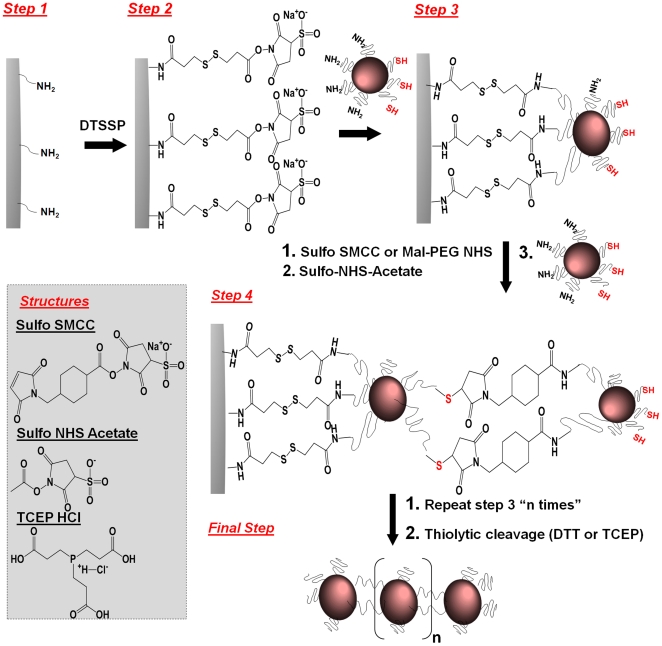
Reaction scheme of the controlled assembly of linear nano-chains from ASC nanospheres using solid-phase chemistry.

Linear nano-chains were assembled from ASC nanospheres on a solid support as shown in Figure 5. The assembly process began with a solid-phase synthesis similar to the preparation of the ASC nanospheres, using CLEAR resin and DTSSP cross-linker (step 1). The first wave of ASC nanospheres was attached onto the solid support (step 2) followed by an excess of sulfo-NHS acetate to block any unreacted amine groups on the solid phase (step 3). The acetylation/blockage is a crucial step to control the 3D structure of the chain. Step 3 also includes the introduction of the heterobifunctional crosslinker sulfo-SMCC followed by the next wave of ASC nanospheres. This process was continued until the desired length of the nano-chain was obtained. A reducing agent, such as DTT or TCEP, was added in the final step to cleave off the final nano-chain from the resin.

FTIR analysis was used to monitor the formation of covalent bonds during the synthesis of the nanochains. Following modification of the resin with the crosslinker DTSSP (step 1 in Figure 5), predominant characteristics of the spectrum included the appearance of IR bands at 525 cm^−1^ (S-S stretching) [29], 1038 cm^−1^ and 1135 cm^−1^ (relatively strong sulfonate resonances) [29], [30], [31], 1790 cm^−1^ and 1730 cm^−1^ (imide resonances) [30], [32], 1650 cm^−1^ (amide I band), 1550 cm^−1^ (weak amide II band) [29], [32], and 3040–3700 cm^−1^ (broad region for N-H, O-H stretching from amide and absorbed water) [29], [32]. The corresponding bands of the sulfonate (indicative of the NHS ester) and imide groups were used to validate the modification in each step. After step 2 of the synthesis, the IR peaks of the sulfonate and imide groups were significantly reduced in the IR spectrum indicating the attachment of the ASC spheres to the resin. The attachment of the crosslinker sulfo-SMCC to the first ASC sphere (step 3) was confirmed due to the appearance of strong sulfonate peaks and imide bands. As a result of the conjugation of the second ASC sphere onto the nanochains (step 4), the corresponding bands of the sulfonate and imides groups were significantly decreased.

The number and size of the ASC spheres used in the assembly can result in nano-chains of different length and aspect ratio. For example, we assembled three different chains consisting of (1) either three consecutive 10 nm spheres (denoted as NC-3×10), or (2) three consecutive 30 nm spheres (denoted as NC-3×30), or (3) two consecutive 30 nm spheres which were sprinkled with 10 nm spheres (denoted as NC-2×30_(10)_). The latter nano-chain was created by attaching two spheres of 30 nm on the solid support and conjugating 10 nm spheres onto the available amines on the sides of the chain.

**Figure 6:**
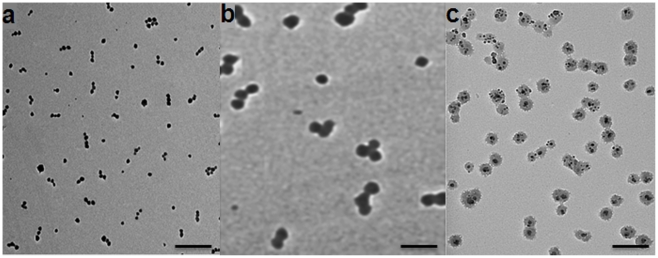
TEM images of linear nano-chains assembled from iron oxide nanospheres with various sizes. (a) Linear chains of three 10 nm iron oxide nanospheres (NC-3×10), (b) Linear chains of three 30 nm iron oxide nanospheres (NC-3×30), and (c) Linear chains of two 30 nm iron oxide nanospheres sprinkled with 10 nm iron oxide nanospheres (NC-2×30_(10)_). Scale bar is 100 nm.

TEM was used to analyze the iron oxide nano-chains. As shown in Figure 6a, NC-3×10 was synthesized in a highly controlled manner. Most of the nano-chains are linear and consist of 3 spheres. Notably, all three classes of nano-chains displayed a similar consistency. Table 1 summarizes the important parameters of each class of nano-chains. In the case of NC-3×10, 3.03 (±0.31) ASC nanospheres per chain were measured via visual inspection of multiple TEM images. Due to the simple and easy removal of unbound spheres in our method, a small number of free nanospheres that were not associated within a nano-chain was observed. The hydrodynamic diameter of NC-3×10 was 39.5 nm, whereas the actual size was essentially the summation of the lengths of its constituting spheres. It should be noted that dynamic light scattering measures an effective diameter based on the diffusion of the particle. Similarly, the characteristics of the other two formulations showed a high degree of uniformity indicating the engineerability and great control of the nano-chain synthesis.

**Table 1:** Summary of the main characteristics of the three different nano-chains.

ID	Hydrodynamic diameter (nm) a	Nanospheres per nanochain b	% nanospheres in nanochains c
NC-3×10	39.5 (±9.1)	3.03 (±0.31)	71.3
NC-3×30	113.5 (±8.5)	2.67 (*±0.78*)	73.0
NC-2×30_(10)_	70.36 (±10.2)	2.55 (±0.51)	87.3

a Hydrodynamic diameter was obtained from DLS measurements (data presented as mean ± standard deviation).

b Values were obtained from visual analysis of TEM images (minimum count was 200 particles; data presented as mean ± standard deviation).

c The number of nanospheres participating in the formation of nano-chains compared to the total number of nanospheres present in the suspension.

It has been reported that iron oxide nanoworms display increased magnetic relaxivity in MRI compared to spheres due to enhanced orientation of the magnetic moments of the different iron oxide cores [10], [33]. Besides the effect of shape, previous studies have reported that clustering of iron oxide cores also significantly increases T2 relaxivity [34], [35]. To evaluate the effect of the geometry on the magnetization, we compared the r2 relaxivity of the NC-3×30 nano-chain to that of its parent 30 nm iron oxide spheres by measuring the transverse (*R*2) relaxation rates at 1.4 Tesla, a typical field strength used in clinical MRI. Notably, the r2 value of the nano-chain is 2.25 times higher than that of its constituting spheres (Table 2).

**Table 2:** Comparison of the T2 relaxivity of a nano-chain to its constituting iron oxide nanospheres.

ID	T2 relaxivity (s^−1^ mM ^−1^)
30 nm spheres	44.87
NC-3×30	101.05

## Discussion

Due to the simplified purification procedure and easy handling of multiple reaction vessels, the solid-phase synthesis technique is a useful tool to control the chemical functionalization of nanoparticle materials [19]. However, due to the size, mobility, and solubility restrictions, solid-phase reactions involving nanoparticles is challenging [19]. The functional group density and swellability of the solid support are important parameters that dictate the number of functional groups available for binding to the nanosphere. We tested various solid supports to obtain ASC nanospheres with high purity and yield. Resins that are highly swellable in water, such as PEGylated resins (CLEAR with amine functional group densities of ∼0.72 mmol/g), were found to be best suited for our application. Similar results were reported by Sung et al. on their synthesis of monofunctionalized gold nanoparticles by Fmoc solid-phase[20]. Yield was observed to be better for nanoparticles prepared using poly(ethylene glycol)acrylamide copolymer (PEGA)-based resin compared to a polystyrene solid support (Wang resin). Similarly, Worden et al. reported a solid-phase place exchange reaction for the synthesis of gold nanoparticles with a single functional group on their surface by controlling the density of thiols (i.e. low enough to be located far apart from each other) on the solid-phase [19]. Both approaches were focused on the preparation of a single functional group on the particle surface. However, our goal was to obtain multiple functional groups of the same type to achieve ASC nanospheres. In addition, our choice of crosslinkers provides flexibility to our synthesis compared to the previously reported “catch and release” mechanism to control the number of functional groups attached to the nanoparticle surface [19], [20], [36]. While cleavage of the nanoparticle from the solid support can be achieved using harsh conditions, such as acidic (trifluoroacetic acid [20]) or basic conditions (ammonia [19], [22]), high temperature [20], [22] or sonication [22], our strategy is performed in aqueous environment and at physiological pH, which are very important for the stability of nanoparticles.

Importantly, this approach of generating chemical asymmetry provides excellent flexibility. For example, it can be observed in Figure 4c that only a very small portion of the surface would be modified (PMA<10%) if larger particles (>80 nm) with the same type and size of coating were used. Taking under consideration however the particle's size, the length of the tether as well as that of the crosslinker, the ASC modification can be controlled to rationally design the nanoparticle's bifunctional surface according to desirable asymmetries.

The controlled asymmetry allowed us to assemble them in a linear orientation with a high degree of uniformity indicating the engineerability and great control of the nano-chain synthesis. While other strategies have resulted in linear chains at the nanoscale [7], [8], [9], [10], [11], they are either appropriate to only one type of material (e.g. iron oxide) or do not control the size very precisely. Our methodology offers exceptional flexibility in synthesizing nano-chains consisting of various types of constituting members (e.g. dendrimers, gold particles) with different functions (e.g. imaging, therapy). One could also envision that the use of longer crosslinkers (e.g. NHS–PEG-Maleimide) can provide controllable spacing between the nanospheres.

In conclusion, we were able to define the topology of two different functional groups on the surface of a nanosphere using solid-phase chemistry. Based on theoretical and experimental work, the surface asymmetry is partially dictated by the size of the nanoparticle. Future work will be focused on adjusting other key parameters (e.g. length of crosslinkers) to obtain an even better control of the asymmetric surface chemistry. Importantly, we demonstrated that the surface asymmetry of the nanospheres facilitates the precise assembly of nano-chains with well-defined structure. Furthermore, the nano-chains exhibited higher magnetic relaxivity than its constituting iron oxide particles. One envisions that this methodology can be adapted for various types of nanomaterials.

## Materials and Methods

### Synthesis and characterization of iron oxide nanospheres with asymmetric surface chemistry

Solid-phase chemistry was used to partially modify the surface functionality of iron oxide nanospheres (Ocean Nanotech LLC, Springdale, AR). As reported by the manufacturer's product specifications sheet and previous publications [37], [38], the iron oxide nanospheres were prepared using iron oxide powder as the iron precursor, oleic acid as the ligand, and octadecene as the solvent. The particles were coated with a triblock polymer consisting of polybutylacrylate segment (hydrophobic), polymethacrylic acid (hydrophilic) and a hydrophobic carbon side chain. Amine-terminated polyethylene glycol polymer was conjugated onto the carboxyl groups of the surface of iron oxide nanoparticles. Various resins with high swellability in water, such as PEGylated resins with different densities of amine functional group, were used as solid support. In a typical experiment, 250 mg of CLEARTM (Cross-Linked Ethoxylate Acrylate Resin) resin (Peptides International Inc, Louisville, KY) was placed in a fritted reactor and was washed and swollen in DMF followed by PBS. To attach nanospheres onto the resin, homobifunctional cleavable cross-linkers such as 3,3′-Dithiobis(sulfosuccinimidylpropionate) (0.32 mmol, DTSSP; Thermo Scientific, Rockford, IL) was introduced and allowed to react for 15 min as shown in Figure 2. After the washing/drying cycle to remove unbound DTSSP, 1 mL of amine functionalized iron oxide nanospheres at 1 mg/mL iron concentration was added and mixed with resin beads. The conjugation reaction was allowed to proceed for 45 min with shaking. The nanosphere-resin complex was filtered and a washing/drying cycle was carried out to remove unbound nanospheres. Tris[2-carboxyethyl] phosphine (1.8 mmol, TCEP), a reducing agent, was added and kept for 45 min to cleave off the nanospheres from the resin. The suspension of iron oxide nanospheres with ASC was collected and dialyzed in 2000 Da MWCO membrane against PBS to remove the excess cleaving reagents.

To evaluate the topology of each functional group, we tagged the amines or the thiols on the nanosphere's surface with an excess of the appropriate gold probes (diameter of 1.4 nm; Nanoprobes, Yaphank, NY) and then obtained transmission electron microscopy (TEM) images. More specifically, we incubated the nanosphere with a 10-molar excess of (1) NHS-functionalized gold probes, (2) maleimide-functionalized gold probes, (3) a mixture of NHS- and maleimide- functionalized gold probes, and (4) non-functionalized gold probes. Each suspension was then dialyzed against PBS using a 100 kDa MW cut-off membrane to remove unbound gold probes. TEM images were obtained using a Tecnai F30 instrument (FEI, Hillsboro, OR) operated at 300 kV. The sample was prepared by dropping 3 µL of the nanosphere suspension onto a 400-mesh formvar carbon-coated copper grid, then the excess solution was blotted with a filter paper and the residual wetting layer was allowed to dry in air. The sizes and zeta potentials of the nanospheres were determined using a ZetaPALS dynamic light scattering system (Brookhaven Instruments, Holtsville, NY). The concentration of iron was determined via ICP-OES (Optima 7000 DV; Perkin-Elmer, Waltham, MA).

### Theoretical analysis of the asymmetry on the nanosphere's surface chemistry

A number of factors govern the interaction of tethered ligands on the surface of a nanosphere and the functional groups on the surface of the resin. Jeppesen et al [27] have shown that the distance of separation between two surfaces plays a significant role in binding of tethered ligand and receptor. As the nanosphere approaches the surface of the resin, the overall energy of the system starts decreasing resulting in the formation of amide bonds between the NHS ester of the resin and the primary amines on the nanosphere. This brings the nanosphere further closer to the resin and at a certain critical distance the overall energy of the system reaches minimum, causing the two surfaces to jump into spontaneous contact. Therefore, the maximum number of bonds is formed between the nanosphere and resin at this critical distance, also referred to as the binding distance. Using a combination of Monte Carlo simulations and diffusion reaction theory, Jeppesen et al [27] have demonstrated that the binding distance can be approximated as a function of the tether size.

To study the effect of nanoparticle diameter and tether length on the surface modification of the nanosphere, the Partially Modified Area (PMA) was calculated similarly to Ghaghada [25], [26]. The PMA of the nanosphere is defined as the fraction of the surface that is capable of binding to the resin. The PMA is a function of tether size, ligand size, and nanosphere size. Figure 4a illustrates the interactions between the nanosphere and the resin. The separation distance between the nanosphere of a radius R and the functional groups on the surface of the resin is given by the binding distance. Therefore, the separation distance is a function of tether length. Furthermore, it is assumed that the tether at the outermost end of the PMA forming a receptor-ligand complex is in a fully extended conformation. Therefore using simple geometry, the active area of a nanosphere can be calculated by the equation: PMA = (2πRH)/(4πR^2^) = H/(2R) = (L-d_B_)/2R, where R is the radius of the nanosphere, and H is given by (L-d_B_). L corresponds to the sum of ligand length and the maximum extended length of the tether, and d_B_ is the binding distance.

### Experimental evaluation of the asymmetric presentation of functional groups on nanospheres

To determine the number of functional groups of each type, the amines on the nanosphere surface were reacted with the Alexa Fluor® 488 NHS ester (Invitrogen, Carlsbad, CA). The amount of Alexa 488 on the surface of the nanospheres was analyzed by the fluorescence intensity using a fluorescence plate reader (Synergy HT; BioTek Instruments, Winooski, VT). In a typical experiment, iron oxide nanospheres with ASC (surface thiols and amines) and their parent iron oxide nanospheres (only surface amines) with different diameters (10, 20, 30 nm) were incubated with 10 molar excess of NHS-functionalized Alexa Fluor® 488 over the surface amines for 2 hours in the dark with stirring. Each suspension was dialyzed against PBS using a 2000 Da MW cut-off membrane to remove unbound fluorescent tags. The purified solutions were pipetted into a 96-well plate and the intensity of the fluorescence signal was measured (excitation 480 nm, emission 520 nm). The exact iron concentration was assessed by ICP-OES after digesting all samples with concentrated HNO_3_ acid. It was converted to particle concentration with the assumption that each particle was made of Fe_3_O_4_ and a 5.2 g/cm^3^ density. The average fluorescence intensity of the nanospheres with ASC was compared to the amine-only-functionalized nanospheres of the same size to obtain the percentage of converted amines to thiols.

### Synthesis of linear nano-chains

Solid-phase chemistry was used to synthesize iron oxide nano-chains. Initially, 250 mg of amine-functionalized CLEAR resin were reacted with DTSSP (0.32 mmol) for 15 min (step 1 in Figure 5). After the washing/drying cycle to remove unbound DTSSP, 1 mL of nanospheres with ASC at 5 µg/mL iron concentration was added and mixed with resin beads (step 2). The conjugation reaction was allowed to proceed for 15 min with shaking. Nanosphere-attached resin was filtered and a washing/drying cycle was carried out to remove unbound particles. An excess amount of sulfo-NHS acetate was introduced and kept for 15 min to block the unreacted amine groups. The heterobifunctional crosslinker sulfosuccinimidyl 4-[N-maleimidomethyl]cyclohexane-1-carboxylate (0.05 mmol, Sulfo-SMCC) was introduced and kept for 15 min. After removing excess crosslinker, the next wave of nanospheres with ASC was introduced (step 3). This process was repeated until the desired length of the nano-chain was obtained (step 4). Reducing agents such as DTT or TCEP were added and kept for 45 min to cleave off the nano-chain from the resin (final step). The nano-chain suspension was collected and dialyzed in 2000 Da MWCO membrane against PBS to remove the excess cleaving reagents. TEM analysis was carried out as described previously. The formation of covalent bonds between solid surface-nanosphere and sphere-sphere was characterized using FTIR spectroscopy. The infrared analyses were obtained using a Thermo Nexus 870 FTIR spectrometer with an attenuated total reflection (ATR) accessory. Spectra over the 4000−500 cm^−1^ range were obtained by the co-addition of 64 scans with a resolution of 4 cm^−1^. The exact iron concentration of the nano-chain formulations was assessed by ICP-OES after digesting all samples with concentrated HNO_3_ acid.

### Relaxation measurements

A Bruker Minispec Analyzer MQ60 was used for T2 measurements at 1.4 Tesla. A total of 300 µL of each sample were placed in a 0.6 mm sample tube and allowed to equilibrate to 40°C. All the measurements were made at 40°C and each measurement was repeated four times to measure variations within the readings. T2 curves were obtained using the instrument's Carr-Purcell-Meiboom-Gill (CPMG) pulse sequence (t_2__cp_mb) with a recycle delay of 20 seconds and 200 data points were collected.

### Data and statistical analysis

To determine the significance of different data sets, one-way ANOVA with post-hoc Bonferroni test was performed (SPSS 15, Chicago, IL). A p-value of less than 0.05 was used to confirm significant differences at the 95% confidence level.
